# Tribological Investigation of Glass Fiber Reinforced Polymer Composites against 52100 Chrome Alloy Steel Based on ELECTRE Decision-Making Method

**DOI:** 10.3390/polym16010062

**Published:** 2023-12-23

**Authors:** Corina Birleanu, Mircea Cioaza, Florina Serdean, Marius Pustan, Paul Bere, Glad Contiu

**Affiliations:** 1 MicroNano Systems Laboratory, Technical University from Cluj-Napoca, 400641 Cluj-Napoca, Romania; corina.barleanu@omt.utcluj.ro (C.B.); marius.pustan@omt.utcluj.ro (M.P.); 2 Department of Manufacturing Engineering, Technical University from Cluj-Napoca, 400641 Cluj-Napoca, Romania; paul.bere@tcm.utcluj.ro (P.B.); glad.contiu@tcm.utcluj.ro (G.C.)

**Keywords:** glass fiber reinforced polymer, tribological properties, coefficient of friction, dry abrasion wear, wear rate, ELECTRE method

## Abstract

Fibers play an important role when studying the tribological behavior of reinforced friction composites. The purpose of the current research is to develop a glass fiber reinforced polymer (GFRP) recipe with improved tribological properties as well as to find the composites with the optimal tribological behavior. A ball-on-disc tribometer was used to perform dry sliding friction testing, the obtained results were then analyzed with the ELECTRE (ELimination Et Choix Traduisant la REalite-elimination and choice translating the reality) method based on a utility matrix having process parameters the applied load, sliding velocity, and weight percentage of the fiber content. The ELECTRE method was chosen to find the optimal tribological parameters, with respect to more performance criteria, because it is one of the best multiple criteria decision-making methods. The optimal combination of parameters for the multi-response characteristics of the investigated friction composite was at an applied load of 10 N, a sliding velocity of 0.1 ms^−1^ and a 54% weight fiber content. The results showed that the addition of glass fiber (GF) content did not considerably improve the tribological behavior of the friction composites. In addition, from the nano focus–optical 3D scanning electron microscopy, images of the friction, tested friction and wear composites, plate formation, fiber-matrix delamination, fiber pull-out, and matrix cracking and damage, various wear mechanisms were identified.

## 1. Introduction

In the past two decades, the incorporation of fillers in GFRP has experienced significant growth, resulting in reduced weight and costs for industrial products. These composites offer numerous benefits, including light weight, affordability, high specific strength, improved insulation, and flexibility [1,2,3]. Investigating the mechanical and tribological properties of fillers and GFRP composites is crucial for optimizing filler parameters such as filler diameter, filler volume, weight fractions, and matrix content.

For many years, fiber-reinforced composites were successfully used in all engineering applications. When fiber and matrix function separately, neither would be able to provide the combined qualities of two or more materials that composite materials produce [1]. The most widely utilized material for producing composites was GFRP composites. Polymer composites reinforced with GF are extensively used in engineering applications due to their superior tribological features [4]. GFRP structures come in several varieties, each with their own special qualities and uses.

A fiber-reinforced composite’s mechanical behavior is primarily determined by the fiber strength and modulus, chemical stability, matrix strength, and interface bonding between the fiber and matrix to facilitate stress transmission [1,5,6,7]. On the other hand, in tribological applications, the composites were put through a variety of conditions, including sliding, rubbing, and rolling against other materials, or against one another. The calculation of the impact of tribological performance considered the load, sliding distance, duration, speed, and sliding conditions [8,9].

Fiber reinforced friction composites are primarily used in brake systems for automobiles and trains where friction and wear are serious problems. The creation of friction layers on friction surfaces and wear detritus between the friction pair are, in general, the two factors that determine the tribological performance of friction materials [2,3,5]. The reinforcing fibers in the brake friction composites show tribofilm formation in the path of tribological stress caused by wear debris compaction. Nevertheless, the formation of the friction layer remains poorly understood, including the associated difficulty and mechanism. The boundary layers, comprising sliding speed, normal load, interface temperature, and variable composition properties, play a crucial role in determining the wear of the friction couple. With the addition of fillers, the GFRP matrix’s wear rate and coefficient of friction were found to be optimal.

Polyamide 6.6 (PA 66) matrix commonly employs GF as one of its extensively utilized reinforcing agents. Typically, these fibers undergo sizing to facilitate strong bonding with the matrix, resulting in a material that exhibits superior mechanical performance. Numerous studies have explored the impact of reinforcement on the mechanical characteristics of this thermoplastic material. They typically demonstrated enhancements in both strength and mechanical properties [10]. Other studies looked at how reinforcement affected the polyamide’s tribological behavior in dry or humid environments. According to S. Srinath and Gnanamoorthy, when nylon 66 is slid against stainless steel, adding roughly 20% weight fraction ratio (wf %) of GF to the material, reduces the specific wear rate by roughly 74% and the coefficient of friction by about 28% (AISI 314) [11]. According to Kim et al., when polyamide was slid against carbon steel (S45C), 30% GF reinforcement increased wear resistance and reduced friction coefficient [12]. Through reciprocating dry sliding wear tests against stainless steel (AISI 431), Byett and Allen demonstrated that 40% GF reinforcing reduced the specific wear rate by about 99% under low load (2 kg) and approximately 76% under high load (10 kg), albeit at the expense of an increase in a coefficient of friction [13].

The most important factors influencing the tribological behavior of wear abrasives include sliding velocity and time, applied force, environmental conditions, radial distance, and reinforcement type, according to more research on functionally graded composite materials (FGMs) [14]. The abrasive wear rates and speed were shown to be inversely related as indicated in [15].

It is important to comprehend the impact of adding reinforcements on the mechanical and tribological properties of polymer composites in order to maximize their use and expand their range of applications. This has led some researchers to perform investigations into the tensile, flexural, friction, and wear characteristics of various GFRP [16,17]. This concern is also followed throughout this work.

In the bibliography, refs [2,18,19,20,21] research investigations were carried out on a composite of unidirectional oriented GF type E epoxy based on the ball-on-ring method to examine the wear and friction properties. The findings indicate that an increase in dry or wet conditions enhances both the friction coefficient and wear rate. 

Numerous tribological applications, such as bearings, gears, wheels, and bushes, have utilized composite materials.

Material and component manufacturers typically possess exclusive knowledge regarding composition and fundamental material properties. Consequently, designers and application engineers find themselves compelled to depend on catalog information and the dispersed literature data. To address this challenge, functional and tribological assessments are conducted using scale models or laboratory setups before implementing a chosen material or component. Once more, determining an appropriate test program remains a significant consideration in these situations. Considering this, the present work is a continuation of the authors’ previous work [4] to obtain a comprehensive analysis for functionally reinforced unidirectional GFRP/epoxy composite materials.

## 2. Materials and Method

### 2.1. Manufacturing and Properties for GFRP Samples

The thermoset polymer matrix used to create the composite material for the experiment was reinforced with a 270 g/m^2^ GF twill fabric [4]. 

The epoxy resin type EPIKOTETM Resin MGS LR 135 (HEXION GmbH, Duisburg, Germany) and EPIKURETM Curing Agent MGS LH136 hardener, with a mix ratio of 100: 35.2 g, constitutes the polymer matrix. These materials are frequently used for processing glass, carbon, or aramid fibers [4].

Six layers of 300 mm × 300 mm, 270 g/m² GF twill fabric was overlaid and impregnated with epoxy resin using the hand lay-up method (wet technology) in a temperature-controlled mold. To get rid of any air bubbles that may have developed between the overlay layers, the entire surface was rolled by hand.

The composite material underwent a heating process within the mold, starting at 80 degrees for 10 min, followed by a subsequent increase to 120 degrees. During this phase, a gradual application of pressure ranging from 1 to 10 tons persisted for 12 h. This combined temperature and pressure treatment effectively eliminated the microscopic bubbles of air and the surplus resin from the composite material. Post heat treatment, the material was allowed to cool within the mold under sustained pressure.

Upon completion of the specified procedure, GFRP materials with dimensions of 300 mm × 300 mm × 2 mm were successfully produced. These materials exhibited weight fraction ratios of 68.5%, 65.3%, and 54%. Subsequently, discs with a diameter of Ø 50 mm were crafted from these materials for experimental purposes.

### 2.2. Mechanical Properties of GFRP Samples

Measurements of tensile, impact, and hardness were used to determine the mechanical characteristics of GFRP. Five specimens of the GFRP materials were examined for their tensile and flexural characteristics using the standard test methods ASTM D638-14 (standard test method for tensile characteristics of plastics) [22] and ASTM D790-03 (standard test methods for flexural properties of unreinforced and reinforced plastics) [23].

A three-point flexural test was performed on the bending samples using an Instron 3366–10 kN (Instron, Norwood, MA, USA) universal testing machine. The specimen was subjected to a load at a testing speed of 2 mm/min until it broke. The average values of the flexural mechanical properties and the standard deviation from all analyzed samples are shown in Table 1.

On an Instron 8801–100 kN (Instron, Norwood, MA, USA) servo-hydraulic testing machine, tensile specimens were tested. Transverse displacement was used to gather the strain data. The tensile mechanical properties and standard deviation for the analyzed specimens are shown in Table 2.

The mechanical characteristics under evaluation of strength, strains, and modulus of elasticity exhibited a high level of reproducibility in the tests, as indicated by the standard deviation. This emphasizes the reliability and significance of the obtained results.

To evaluate the mechanical properties, particularly the hardness, of the GFRP disc, a Mitutoyo HR-430 Series Digital Rockwell Hardness Tester (Mitutoyo Europe GmbH) was employed. Three samples underwent testing with a 5% margin of allowable error for density and hardness measurement, adhering to standard industry practices. From the composite material a specimen of 30 mm × 30 mm dimensions was extracted. The hardness testing took place in a laboratory environment under natural atmospheric conditions. Each specimen underwent indentation at a minimum distance of 3 mm from both its periphery and any other indentation. Indentation was exclusively performed on smooth surfaces coated with polyester resin. Each specimen underwent five readings, and the results were duly documented. 

The Rockwell Superficial Hardness Scale 30T-describes a specific configuration for conducting Rockwell superficial hardness tests on materials with a particular scale, indenter type (diamond ball), indenter size (1.5875 mm), and preliminary test force (294.2 N). The hardness value obtained from this test provides information about the material’s hardness characteristics, especially useful for unhardened materials, such as metals softer than hardened steel, or where shallow indentations are desired. Hardness values derived from these measurements are detailed in Table 3.

The R_a_ roughness parameter was assessed following ISO GPS standards, employing a Gaussian filter with a sampling length of 0.8 mm and an evaluation length of 3.2 mm. The ISR-C300 INSIZE Detachable Probe Rugometer was utilized to measure the surface roughness of the C_1_–C_3_ discs. Roughness tests were performed along the fiber direction, perpendicular to the fiber and at 45° to the fiber direction, and the average roughness parameter R_a_ value presented in Table 3 is an average of these measurements.

Table 4 displays the roughness values of the ball, providing valuable information for the development of accurate wear models and the execution of predictive tribological assessments.

### 2.3. The Ball Sample

A bearing ball composed of 52100 chrome alloy steel with diameter of 12.7 mm provided by RKB Bearing Industries Group is what was used as a counterpart for the wear tests. This type of ball is widely used in the industry for ball and roller bearings because of their exceptional surface quality, superior wear resistance, hardness, and high load capacity.

Table 4 contains details regarding the mechanical properties of the ball utilized in this research. The details of the ball properties were provided by the supplier.

### 2.4. ELECTRE Decision-Making Method

When a GFRP with improved tribological properties is required for a certain engineering application, the choice of the material to be used can be made with respect to several criteria, such as maximum friction coefficient or minimum specific wear rate. The choice between the material recipes tested for this paper was made using the ELECTRE method, which has a non-compensatory nature and employs a simple logic and a refined computational process. This method was previously used by some of the authors to assist the decision making between several water filters available to customers with respect to several criteria [21].

The proposed approach makes it easier to select the best option and provides a rational basis for estimating the benefits of several potential courses of action [21]. The best option is determined based on several decisional criteria and their assigned importance weights, ensuring that the utility provided by a material recipe is computed according to the economic value of the material recipe option with respect to the chosen decisional criteria.

The basis for the utility computation is presented in matrix form and it is encompassed in Table 5; one of the advantages of using this method being that it fully utilizes the information contained in the decision matrix.

First, the values o_ij_ corresponding to each material recipe (option) with respect to each considered criterion are transformed in utilities u_ij_ (that are encompassed in a table like Table 5). Afterwards, for each criterion Cr_j_, j = 1 − n, the minimum value o_jmin_ and the maximum value o_jmax_ are determined. Within each column, the utility value 1 is given to the “best” option and the utility value 0 to the “worst” option. Regarding the other values, their utilities u_ij_ are computed in each column as follows:

- for a maximum criterion:

(1) $$u_{\mathrm{ij}}=\frac{o_{\mathrm{ij}}\;-\;o_{\mathrm{jmin}}}{o_{\mathrm{jmax}}\;-\;o_{\mathrm{jmin}}}$$

- for a minimum criterion:

(2) $$u_{\mathrm{ij}}=\frac{o_{\mathrm{jmax}}\;-\;o_{\mathrm{ij}}}{o_{\mathrm{jmax}}\;-\;o_{\mathrm{jmin}}}$$

The following step is to find the concordance indices between two material recipe options, based on the equation:

(3) $$C\left(O_{g}{,O}_{h}\right)=\frac{\sum w_{j}^{*}}{w_{1}+...{+w}_{n}}$$

where $\sum w_{j}^{*}$ is the sum of the importance weights of the criteria for which the following restriction exists: $U\left(O_{g}\right)\geq U\left(O_{h}\right)$

Afterwards, the discordance indices are determined:

(4) $$D\left(O_{g}{,O}_{h}\right)=\left\{\begin{array}{l} 0,\;\;\;\;\mathrm{if}\;U\left(O_{g}\right)\geq U\left(O_{h}\right)\\ 1/\alpha \;\max\left|U\left(O_{g}\right)-U\left(O_{h}\right)\right|,\;\mathrm{otherwise} \end{array}\right.$$

where: α = maximum gap between minimum and maximum utility.

In the end, the classification of the material recipes considered as options is based on the difference between the concordance and discordance indices, thus the ELECTRE method offered a good assessment of the advantages of one material recipe option over another, while avoiding compensating for a very poor criterion value with good values on other criteria. 

## 3. Experimental Method and Device

**Figure 1:**
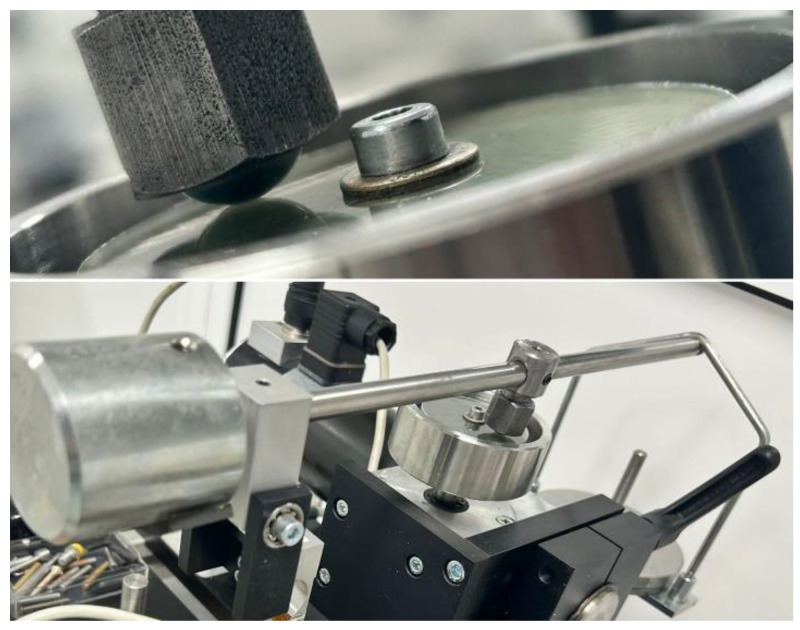
A modified ball-on-disc tribometer.

The wear tests were carried out on a ball-on-disc tribometer (Figure 1), in an ISO 6 clean room (Class 1000 cleanroom). This is a soft- or hard-sided wall manufactured structure that utilizes HEPA filtration systems to maintain air cleanliness levels of a maximum of 1000 particles (≥0.5 µm) per cubic meter of inside air. The temperature provided is 22 °C, a relative humidity of 45%, dry lubrication conditions, and a duration of sliding of 120 min.

Friction and sliding wear tests were carried out using a ball-on-disk tribological machine. Disc diameters of 50 mm with thicknesses of 2 mm were used for all sets of experiments. The pin was substituted with a bearing ball featuring a diameter of 12.7 mm, customized to fit the clamping system.

For each test, a fresh ball and disc were employed, and before commencing the test, they were cleaned and wiped dry with a technical cleaner. The test extended for 120 min, during which the friction coefficient was consistently monitored to identify the running-in friction regime and the friction coefficient in the steady-state regime. The loads and sliding velocities ranged from 10 N to 30 N and from 0.1 m/s to 0.36 m/s, respectively. A full distance of 0.72 km, 1.81 km to 2.59 km was achieved with the three selected speeds at r = 16 mm for the distance between the axis of the fixed ball and the rotation disc. Throughout the test, temperature, wear, and friction force were consistently monitored with a precision tolerance of 2–3%. The ball or disc’s wear loss was quantifiably assessed using surface profiling and microscopic analysis. The conditions of the friction tests related to the ELECTRE decision-making method were carried out according to the control parameters summarized in Table 6.

To enhance the reliability of the test outcomes, the experiment was iterated three times, and the average value was adopted. To indicate how spread the data are around the mean value, error bars were used, representing the corresponding standard error of the mean.

For weight loss attributable to wear, an analytical microbalance with an accuracy of 0.1 mg was used to measure the weight loss of both the ball and the disc.

**Figure 2:**
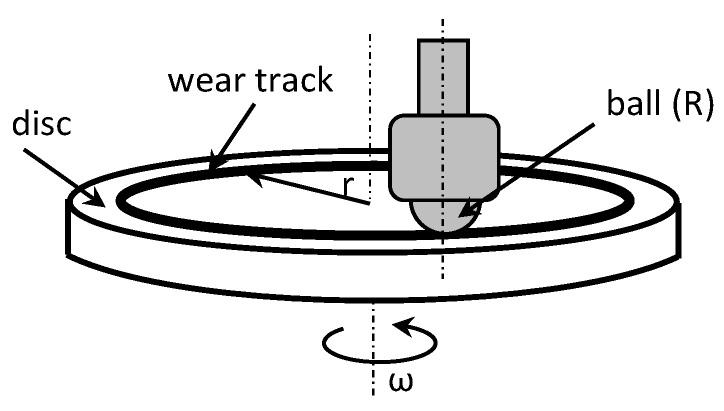
Contact configuration for the friction test (wear track model) [1].

In instances where distinctions in weight loss attributable to wear were indiscernible, for deeper insights into the wear phenomenon and the mechanisms of material removal, optical 3D microscopy (OM) was employed to scrutinize the worn surfaces of the ball and the track surface of the disc after each test. Surface modifications were explored using nano focus–optical 3D microscopy, employing advanced μsurf technology for precise 3D surface measurements. This methodology relies on continuous focus variation technology with fixed focal length objectives, with a scanning target set at 10× zoom. From the scanned surface, the radial layers were isolated and analyzed to gain insights into the surface characteristics. The wear volume of the samples was computed by measuring the wear width and depth using the profilometer as depicted in Figure 2. These calculations were executed using empirical mathematical equations, assuming the validity of the ideal ball geometry that shapes the wear scars.

The wear rate (K, mm³/Nm) researched in this paper was determined using the following equation:

(5) $$K=\frac{m}{L_{sliding}\cdot F\cdot \rho }=\frac{V}{L_{sliding}\cdot F}$$

where m is the loss in mass (mg), L*_sliding_* is the sliding distance (m), F is the applied load (N), V is the loss volume (mm^3^), r is the distance between the axis of the fixed ball and the rotation ring, and ρ is the density of material samples (g/cm^3^). To minimize data variability, three duplicate sliding tests were conducted in this research, and the mean values of the coefficient of friction and wear rate were employed for analysis.

The highest temperature under typical dry sliding conditions occurs in the microcontact zone itself between the two sliding surfaces. A Flir E30 infrared camera, equipped with a 160 × 120 IR pixel resolution, was employed to monitor temperature variations throughout the tests. The camera boasts a temperature range spanning from −20 to 250 °C, coupled with an impressive thermal sensitivity of 100 mK, or less than 0.1 °C.

## 4. Experimental Results and Discussion

### 4.1. Sliding Speed Effect and Applied Load on Friction and Wear

In this phase of the study, three material couplers were tested consisting of a GFRP composite disc C_1_, C_2_, and C_3_, and a ball made of 52100 chrome alloy steel. Because of their remarkable qualities, these materials are widely used in technical applications. The 52100 chrome alloy steel is a high-carbon, chromium-containing alloy that is widely used as an engineering material, particularly in applications where high strength, wear resistance, and durability are required. It is known for its excellent hardness, toughness, and fatigue resistance, making it suitable for a variety of applications, including bearings, cutting tools, automotive components, and other machinery parts. GFRP composites find wide-ranging applications in various industries due to their desirable properties. GFRP and chrome alloy steel dry friction couplings are used in various engineering applications where a reliable and durable connection between two rotating components is required. These couplings are designed to transmit torque from one component to another without the need for lubrication, making them suitable for environments where the use of lubricants is not feasible or desirable [9].

The tribological behavior of the 52100 chrome alloy steel and GFRP composites differs significantly due to their unique material properties. Factors such as friction, wear resistance, lubrication, and environmental sensitivity play crucial roles in determining their suitability for specific applications. The choice between these materials would ultimately depend on the specific requirements and conditions of the intended application.

Tests were conducted with a load of 10, 20, and 30 N at three different sliding speeds (0.1, 0.25, and 0.36 m/s) to ascertain the behavior of the materials.

**Figure 3:**
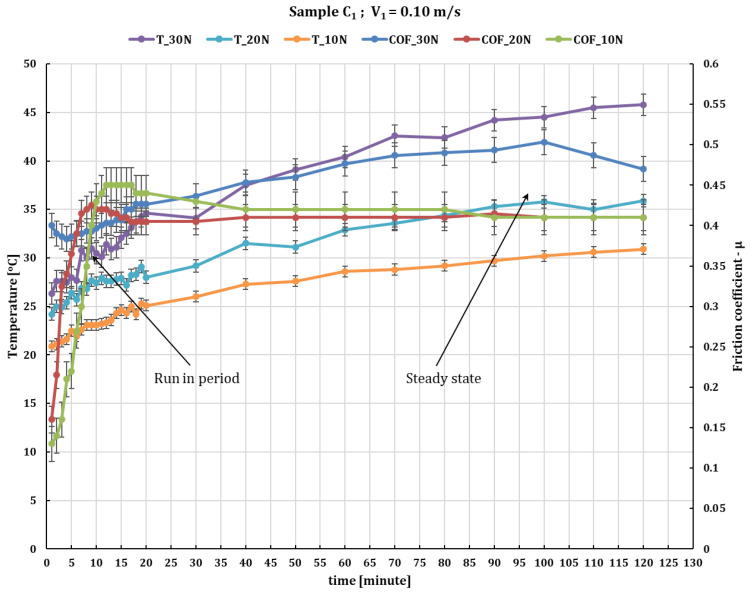
The variation in friction coefficient of C_1_ composite against 52100 chrome alloy steel versus normal loads at test time (120 min) and peripheral speed 0.1 m/s.

Figure 3 illustrates the impact of normal loads at 0.1 m/s sliding speed on the dry wear behavior of C_1_ composite in comparison to 52100 chrome steel. At the same time, the temperature was monitored. Figure 3 shows that increasing the normal load increases the stationary coefficient of friction (obtained in the steady state after run-in period) reaching a value of 0.48 at a force of 30 N and 0.43 at a force of 10 N. In addition, the temperature reached up to 45–48 °C at a force of 30 N after 120 min of testing and around 32 °C at a force of 10 N. 

**Figure 4:**
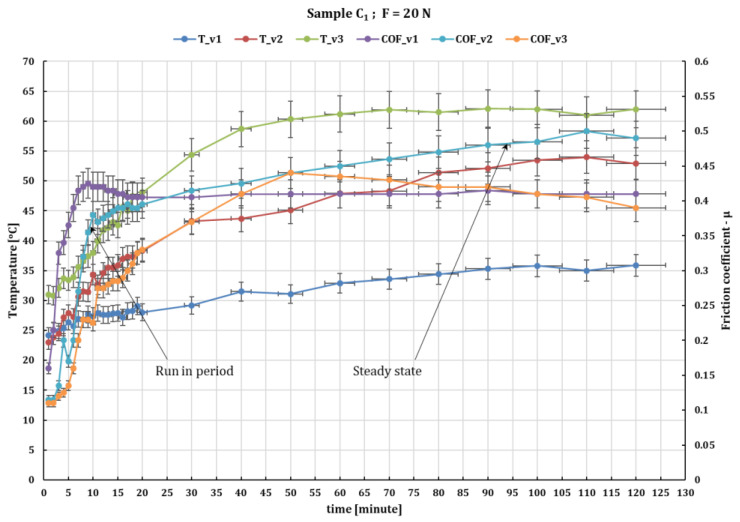
The variation in the values of the friction coefficient of C_1_ composite against 52100 chrome alloy steel versus peripheral speeds at test time (120 min) and loaded with 20 N.

Figure 4 shows that with increasing the speed to 0.36 m/s for composite C_1_ under the same test conditions, the value of the friction coefficient decreases to 0.34 at a force of 20 N, and remains within about the same parameters of values of 0.47 for a force of 30 N. In this case, the temperature in the friction coupling reaches after 120 min of testing 62–63 °C at 20 N load and 88–90 °C at 30 N load. In terms of temperature variation in the C_3_/ball friction coupling the values are quite close to the other experiments with 2–3 degrees higher than in the other experiments. 

In such a tribological system, the decrease in friction coefficient with increasing speed can have several possible explanations, namely, at higher sliding speeds, there is an increase in the generation of heat due to friction. This heat can lead to thermal softening of the polymer, reducing its effective hardness and promoting a decrease in friction.

As the sliding speed increases, the abrasive wear may become less pronounced. This is because at higher speeds, there is less time for the abrasive particles to embed themselves in the polymer and steel surfaces. Sliding action at higher speeds can favor the formation of thin oxide layers on the surface. These oxide layers can act as a form of solid lubrication, reducing direct metal-polymer contact and decreasing friction. The dynamic nature of the sliding interface at higher speeds can influence the surface microstructure of both the polymer and the chromium alloy steel. This dynamic response can lead to changes in topography, reducing grip, and friction.

In some cases, increasing the sliding speed can reduce the sliding behavior, where the surfaces stick shortly before sliding. A reduction in stick-slip can contribute to a smoother sliding motion and a decrease in the overall coefficient of friction.

**Figure 5:**
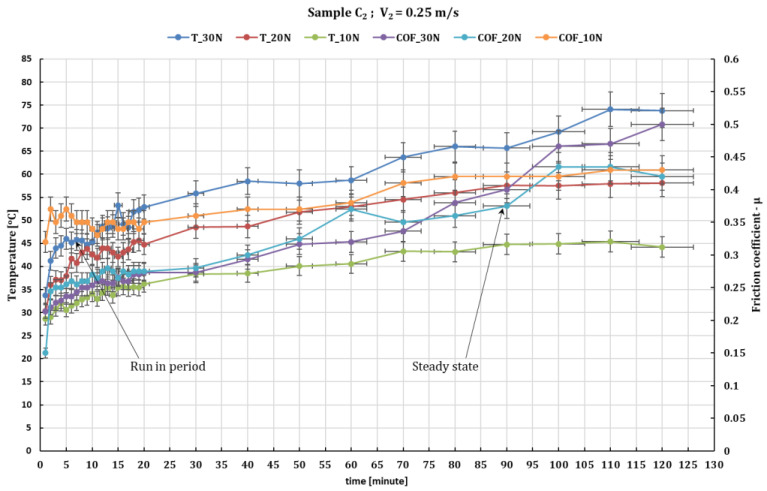
The fluctuation in the values of the friction coefficient of C_2_ composite against 52100 chrome alloy steel versus normal loads at test time (120 min) and peripheral speed 0.25 m/s.

**Figure 6:**
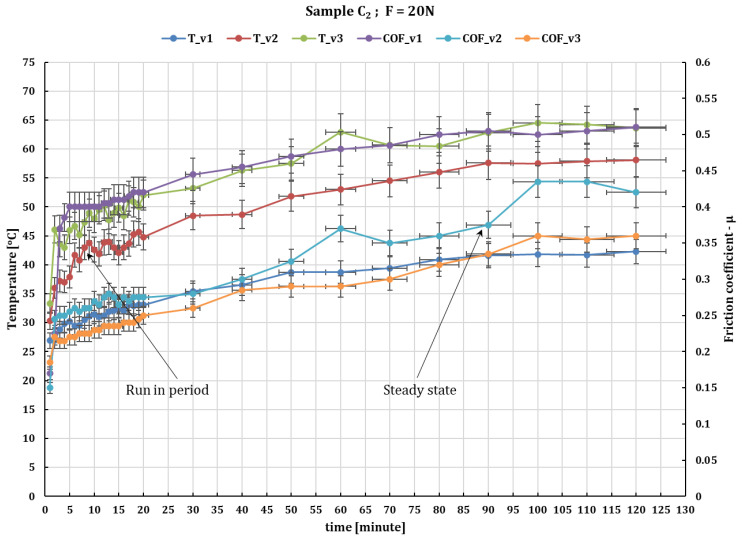
The fluctuation in the values of the friction coefficient of C_2_ composite against 52100 chrome alloy steel versus peripheral speeds at test time (120 min) and loaded with 20 N.

Figure 5 and Figure 6 show the behavior of sample C_2_ under the same test conditions, showing a similar behavior but with a higher coefficient of friction variation. This, with increasing the normal load, increases the stationary coefficient of friction (obtained in the steady state after run-in period), reaching a value of 0.49 at a force of 30 N and 0.5 at a force of 10 N. At a peripheral speed of 0.1 m/s the temperature reaches somewhere between 36 °C and 46 °C for forces of 10 to 30 N and at a speed of 0.36 m/s it reaches 54 °C to 92 °C for forces of 10 to 30 N.

**Figure 7:**
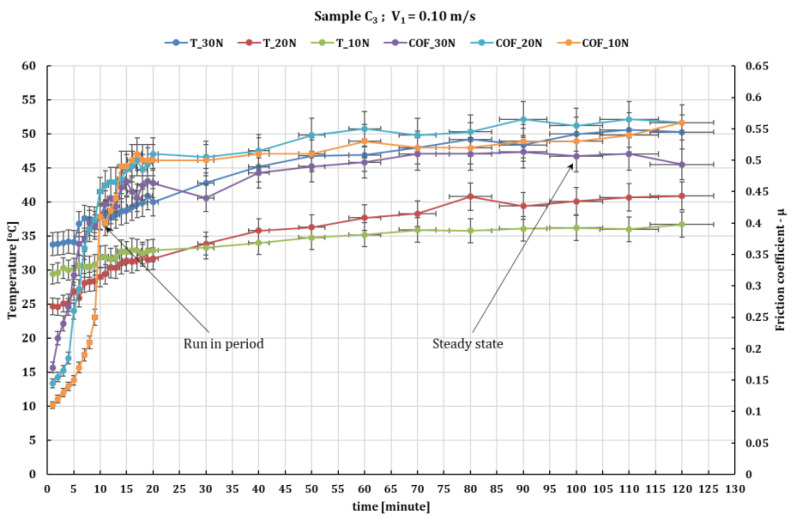
The fluctuation in the values of the friction coefficient of C_3_ composite against 52100 chrome alloy steel versus normal loads at test time (120 min) and peripheral speed 0.1 m/s.

**Figure 8:**
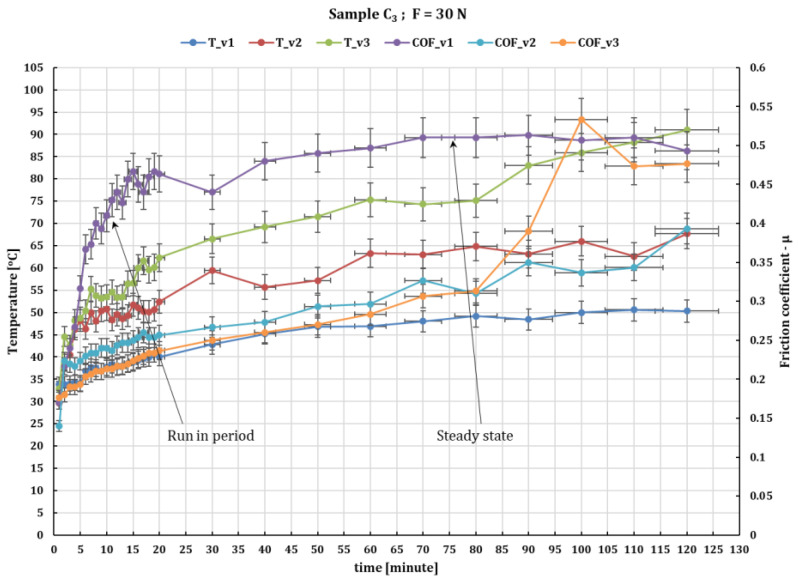
The fluctuation in the values of the friction coefficient of C_3_ composite against 52100 chrome alloy steel versus peripheral speeds at test time (120 min) and loaded with 30 N.

For sample C_3_, which has a content of 54 wf % GF, the results are shown in Figure 7 and Figure 8 that with increasing sliding velocity at a force of 10 N, the friction coefficient decreases from 0.54 to 0.33; for a force of 20 N, the decrease is from 0.54 to 0.37, and at 0.36 m/s sliding velocity for loading from 10 to 30 N, the friction coefficient decreases but within a rather small limit from 0.51 to 0.49.

In terms of temperature variation in the C_3_/ball friction coupling, the values are quite close to the other experiments with 2–3 degrees higher than in the other experiments.

Elevating the proportion of GF diminishes the stationary coefficient of friction, as determined during the stable phase following the initial run-in period. These findings diverge from the observations made by Byett and Allen [13] in the context of reciprocating sliding contact with stainless steel. Conversely, they align with the outcomes reported by other researchers [10,11,12,16] in scenarios involving sliding against stainless steel or carbon steel, or in cases of rolling–sliding contact with identical materials.

On the other hand, the generation of heat due to friction has the potential to induce a plastic state on the contact surface, with early wear debris filling the pores. Under elevated operational conditions, these occurrences might contribute to a decrease in frictional forces. Additionally, heightened levels of heat can foster chemical reactions between the environment and the contact surface, leading to the formation of a robust oxide coating. This coating, serving as a restraint to prevent material removal, can elevate the coefficient of friction. Once the oxide layer attains a critical speed, it detaches from the surface due to increased speed, initiating the material removing process from the surface in contact. Consequently, surpassing this speed threshold results in a decline in the frictional coefficient.

The overall structure and composition of the composite material plays a crucial role. The interaction between the GF and the matrix material, as well as the overall microstructure, can influence frictional behavior. In addition, GF often has a smoother surface compared to other components in the composite. A smoother surface can reduce the adhesion between the surfaces in contact, leading to a lower coefficient of friction. Therefore, a smoother surface reduces adhesion between contacting surfaces. As adhesion decreases, friction between surfaces decreases. The less friction, the less force is needed to move one surface past the other. The presence of glass fibers can act as abrasives that modify the surface characteristics during sliding. This abrasive effect may contribute to a reduction in friction, particularly during the run-in period.

For sample C_3_ at a load of 30 N and at a sliding velocity of 0.36 m/s, heightened levels of heat can foster chemical reactions between the environment and the contact surface from GF composite, leading to the formation of an oxide coating. Also observed was a sudden increase in the coefficient of friction (COF) until 0.55 and a temperature around 93–95 °C after 90 min of rubbing (Figure 8).

**Figure 9:**
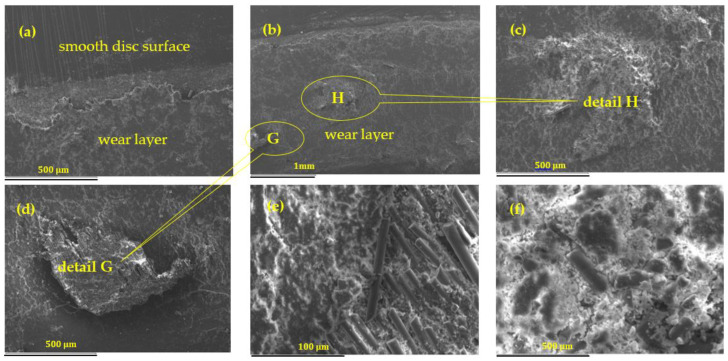
SEM Images of wear track on sample C_3_ after 120 min of operation at 30 N load and 0.36 m/s sliding velocity accompanied by thermal effect. (**a**) At the boundary between the wear layer and the unworn parts; (**b**) view of the worn annular surfaces; (**c**,**d**) accumulation of debris and wear particles on the rubbing surfaces; (**e**,**f**) surface detail of the polymer composite material in the worn area-matrix and glass fibers fragments.

Detailed SEM investigations of the sliding friction wear behavior of GFRP sample C_3_ after 120 min of operation at 30 N load and 0.36 m/s sliding velocity are shown in Figure 9a–f. The observed temperature rise to 93–95 °C falls within a range where oxidation reactions can be accelerated. The sudden increase in the friction coefficient could be an indication of changes in the contact surfaces. Pins made of chromium alloy steel can undergo passivation, a process whereby a more stable and protective chromium oxide layer is formed on the surface. This layer can influence the friction behavior and may cause an initial increase in the coefficient of friction. High heat levels could also lead to chemical reactions between the glass fiber composite and the chromium alloy steel. If any component in the composite is susceptible to oxidation or chemical reactions at high temperatures, this could contribute to the observed changes in friction behavior.

On the other hand, frictional action between the GF composite and the chromium alloy steel may lead to material transfer between the two surfaces. This material transfer, combined with high temperatures, can influence the formation of oxide layers on surfaces.

Increasing temperature causes the soft epoxy resin matrix to soften, forming several spots on the matrix surface, which peel off as the friction composites slide on the counterface. Furthermore, softening of the epoxy resin matrix diminishes the fiber-matrix adhesion and determines if the fiber is to withdraw from the matrix. Hence, with an increase in sliding speed, there is a decrease in the friction coefficient. Table 7 validates that lower speeds correspond to higher values of the friction coefficient. Conversely, the μ value decreases as the sliding velocity increases. Additionally, at lower sliding velocities, the worn surface of the composite friction materials seems smooth, exhibiting limited formation of a primary plateau and fewer wear traces.

Nevertheless, according to Figure 9b, for higher values of the sliding velocity, the surface becomes rough and damaged. Primary plateaus at specific sites are formed by adherent fibers, crucial in halting the movement of fine wear particles at the interface (marked H), resulting in the creation of loose granular films. These loose wear small particles predominately consist of polymeric elements, etc. (marked G), aggregating under increased pressure and temperature during braking. This aggregation leads to the formation of secondary plateaus and the development of dense films. According to the literature, primary platelets could bear loads as well as stimulate friction, while secondary platelets deteriorate the same [5]. For large values of the sliding velocity, the debris particles in the counterface undergo increased impact due to recurring loading on the friction surface. Therefore, this impact loading results in frictional thrust and constrained vibration and jolting at the friction interface, which leads to greater detachment and cracking of the hybrid fibers. The abrasive effect of these hybrid fibers on the sliding interface raises the wear rate of the friction composites by deteriorating the friction film of the counterface with the increase in the sliding speed.

The tribofilm surface created for lower values of the velocity appears smooth, while the one which is created for greater values of the sliding velocity is rough. Therefore, a smaller value of wear rate can be noticed for smaller values of the sliding velocity.

There are several factors at play:

- On one side, the accumulation of debris: debris and wear particles may accumulate on the rubbing surfaces as the rubbing continues. This accumulation can lead to an increase in friction as the debris interferes with the smooth movement of the surfaces;
- And on the other hand, the thermal effects: prolonged rubbing generates heat due to friction. The temperature increase can influence the material properties, potentially leading to changes in the COF. This is especially true if the temperature reaches a point where it causes alterations in the material’s structure or induces other thermal effects.

It is important to consider the specific conditions of the rubbing experiment, including the applied load, speed, and environmental factors. Additionally, analyzing the material composition and structure before and after the 90-min mark may provide insights into the observed changes. Experimental data and analysis of the tribological properties of the specific fiberglass composites involved would be essential to pinpoint the exact reasons for the observed increase in COF and temperature.

As the sliding speed increases, the friction coefficient of the two materials lowers. Because wear particles are present on the sliding surface, both the wear rate and friction decrease with increasing velocity. With increasing velocity, both GFRP and chrome alloy steel experience an increase in mass loss. The color changes in the 3D graph indicate that delamination wear is the predominant wear mechanism for alloy steel.

The increase in COF with the increase in the normal load (from 10 N to 30 N) can be caused by multiple factors. One of the potential explanations is that the elevated normal loads determined an increase in the contact pressure between the sample and the counterpart, which led to an increase in friction. This phenomenon occurs because the contact area between the two materials expanded with the normal load, causing an increase in the pressure between the surfaces. Other contributing factor could be the sample surface deformation. In the research conducted by Ze-Kun Zhao et al. [18] to assess the wear performance of GF and carbon fiber composites, the results indicated that carbon fiber reinforced composites displayed a lower friction coefficient(~0.45 to 0.35) in contrast to GFRP (~0.45 to 0.65).

**Figure 10:**
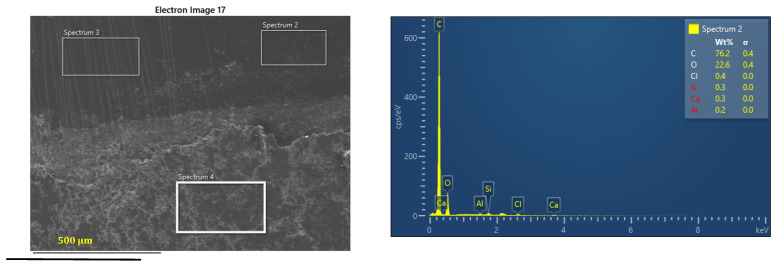

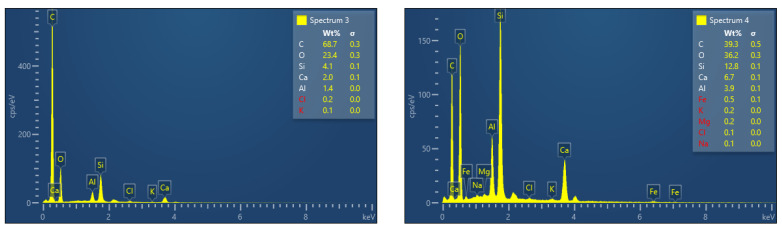
SEM and EDS analysis of worn and unworn surface of the sample C_3_ after 120 min of operation at 30 N load and 0.36 m/s sliding velocity.

The composites were tested for different loads and SEM analysis were performed on their worn surfaces. In Figure 10 it can be seen that for an applied load of 30 N, the hard-wearing debris particles and patches comprise a shear deformed epoxy resin matrix containing broken tiny fibers and wear particles from the countersurface. During wear, several iron particles in the metal counterface intermingle with small broken fiber elements on the friction surface, leading to the creation of tribofilms.

Using elemental EDS quantification of the elements allows us to provide a qualitative analysis of the elements present on the worn and unworn surfaces of glass fiber reinforced composite discs after tribological testing (Figure 10). Elements such as Si, Al, and Ca are found in the worn area in significant percentages compared to the unworn area. In addition, the weight percentage of C decreased to almost half.

These occurring elements suggest the formation of a stable tribofilm in the wear track. The above observation is supported by the change in the friction coefficient from the run-in period to the steady state.

### 4.2. Wear Pattern and 3D Optical Scan of Used Ball and GFRP Specimens

Employing an analytical microbalance with a precision of 0.1 mg, the weight loss of both the ball and the disc served as the basis for wear measurement. With this balance, the weight loss of the chromium alloy balls was negligible, and for the disc in all experimental trials the measured loss was in the range of 0.001–0.030 g and it increases with sliding speed and applied force. Therefore, for the calculation of the wear rate (K, mm³/Nm), the results obtained by scanning with 3D Optical Scan were used. 

**Figure 11:**
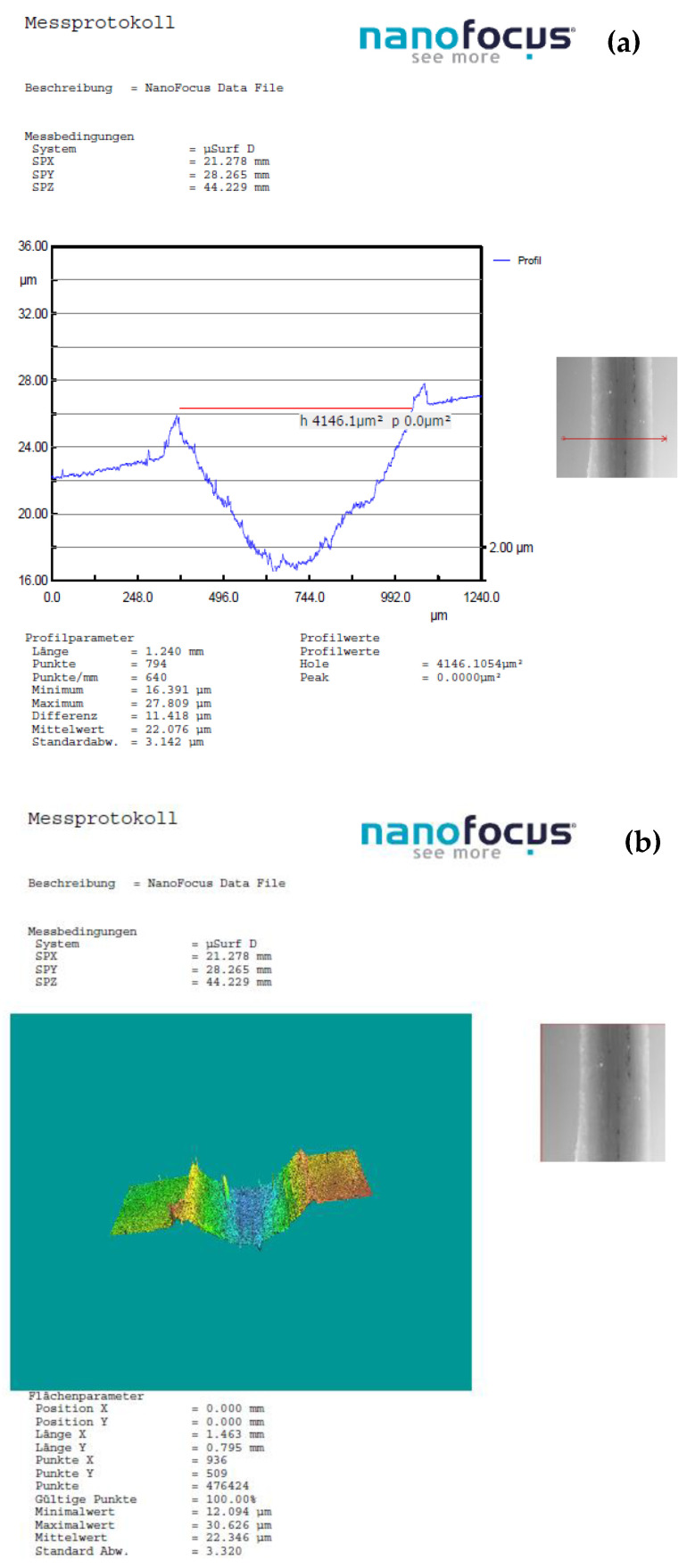
(**a**) The worn surfaces morphologies of C_3_ after friction experiment dry-lubricated under Fn = 10 N, v1 = 0.1 m/s^−1^, duration of 120 min, chrome alloy steel ball contact; (**b**) wear surface profile curve.

**Figure 12:**
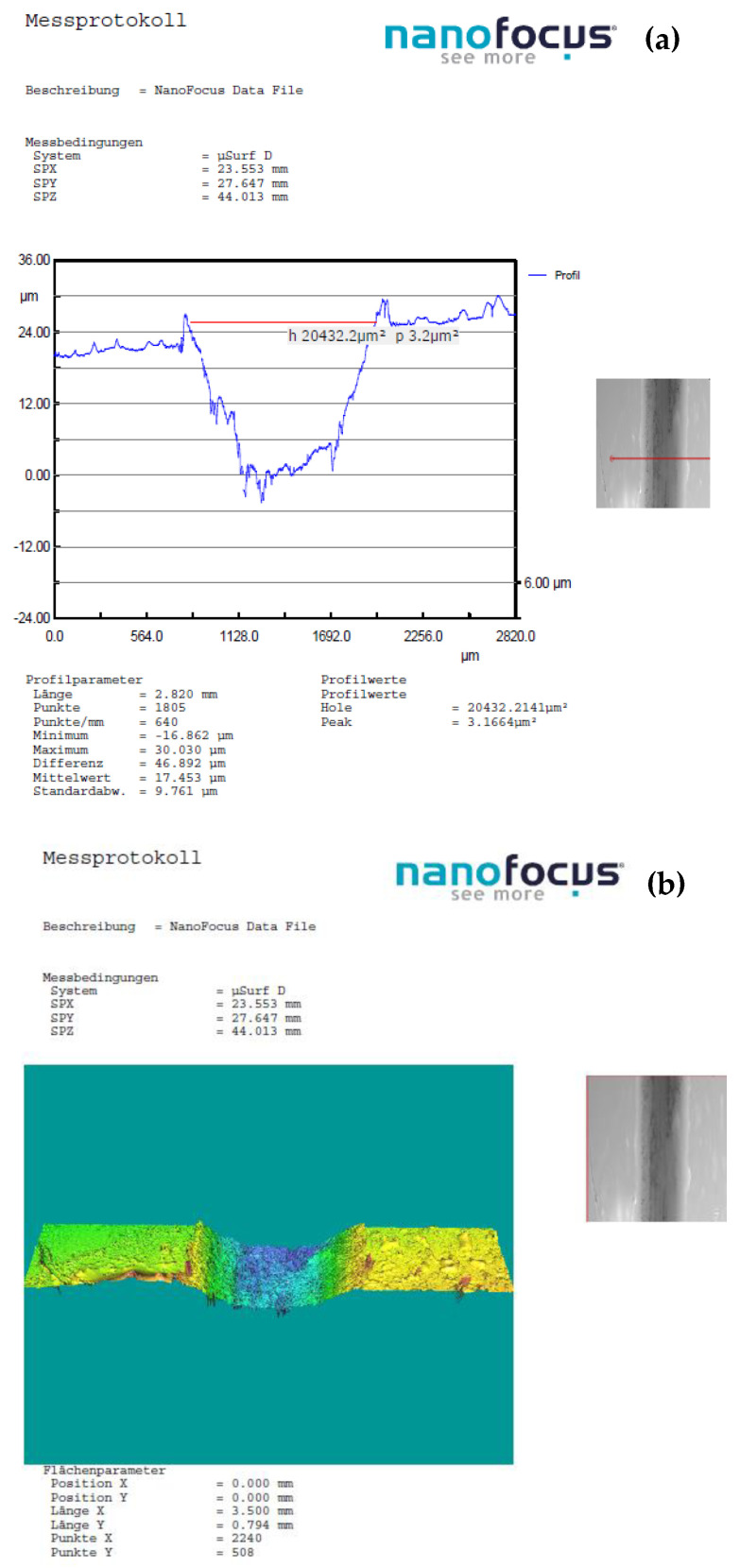
(**a**) The worn surfaces morphologies of C_3_ after friction experiment dry-lubricated under Fn = 20 N, v1 = 0.1 m/s^−1^, duration of 120 min, chrome alloy steel ball contact; (**b**) wear surface profile curve.

**Figure 13:**
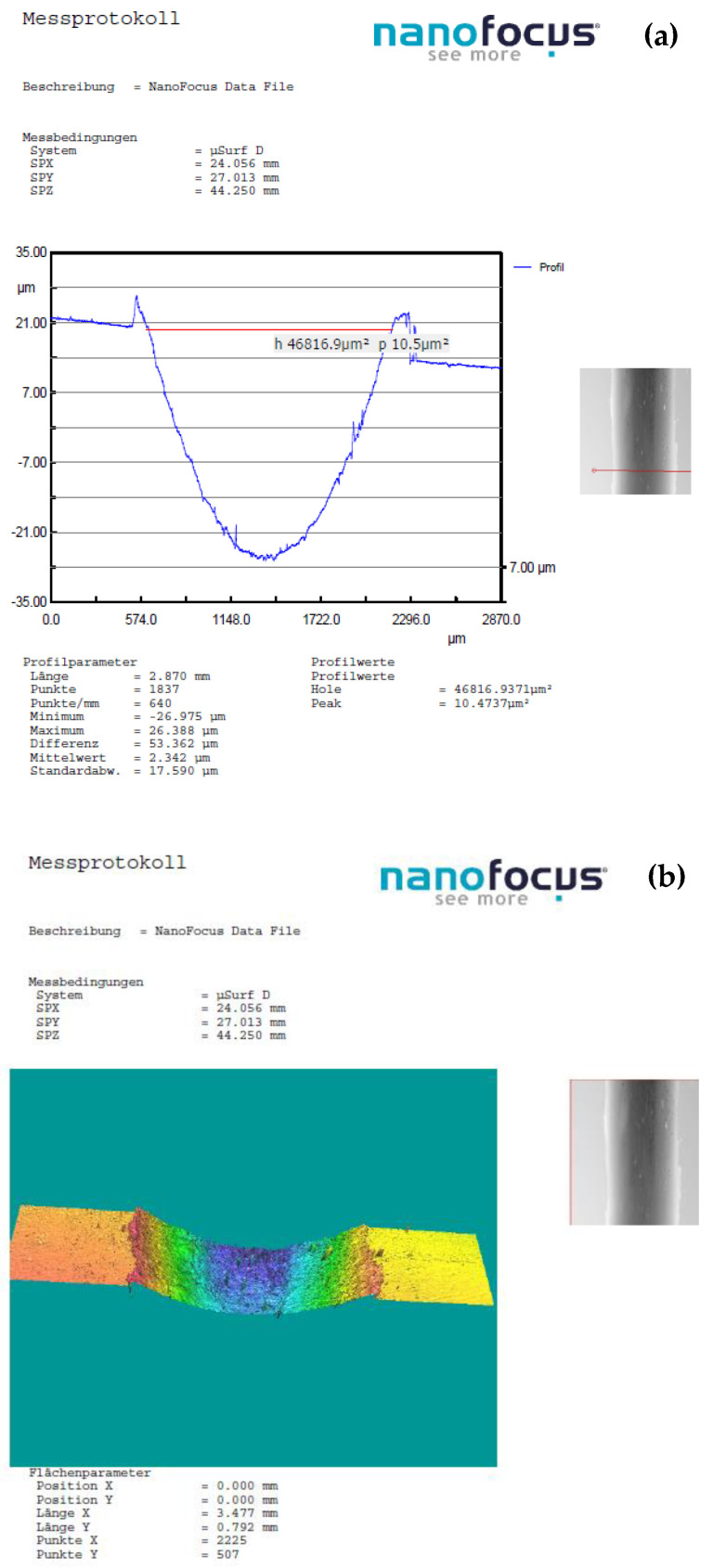
(**a**) The worn surfaces morphologies of C_3_ after friction experiment dry-lubricated under F_n_ = 30 N, v_1_ = 0.1 m/s^−1^, duration of 120 min, chrome alloy steel ball contact; (**b**) wear surface profile curve.

**Figure 14:**
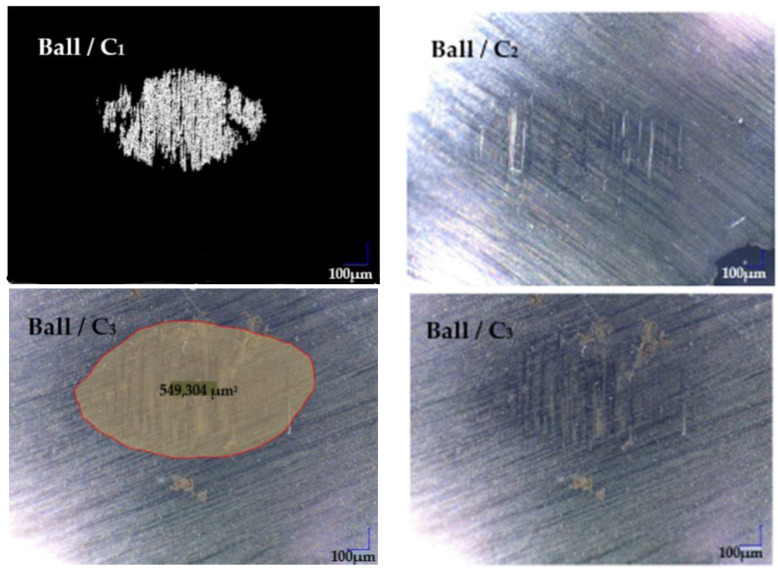
Wear marks of Chrome alloy steel ball against C_1_–C_3_ for 0.1 m/s sliding speeds at 10 N loaded.

Figure 11, Figure 12, Figure 13 and Figure 14 display the 3D optical images and wear pattern of worn-out 52100 chrome alloy steel and GFRP specimens. In the context of solid metal, the extent of wear loss is contingent upon the material density and hardness. The wear rate range for the composite disc, determined through experimental results obtained by profilometry, is presented in Table 7 for three different sliding velocities and various applied normal force values.

It was found that the wear marks of the chrome alloy steel balls show only small scratches on the surface and volumetric wear cannot be measured (Figure 9). An attempt was made to estimate a measurement of the scratched area, obtaining a maximum of 0.5 mm^2^ without being able to measure any wear depth. The observation of small scratches on the surface of balls after 120 min of operation, with no measurable volumetric wear when in contact with a GFRP disc, could be explained by several factors:

- Chromium alloy steel is known for its hardness and wear resistance. The surface of the steel balls is sufficiently hard; it may resist deformation and wear, leading to only minor scratches. The material may also have a good balance of hardness and toughness, allowing it to absorb and resist wear without significant damage;
- The properties of the chrome alloy steel are compatible with the glass fiber composite, resulting in reduced wear. This could include similar coefficients of thermal expansion, which can help minimize thermal stresses;
- And the thermal reactions occurring at the interface between the steel and composite materials may form protective layers that reduce wear (Figure 10).

Various factors, such as sliding speed, could influence the wear rate K, between GFRP and chrome alloy steel. Generally, a decrease in the values of the wear rate between GFRP and chrome alloy steel can be detected with the increase in the sliding velocity.

A plausible explanation for this trend is the impact of sliding velocity on the creation of a transfer layer. When the sliding speeds are low, the interaction between the ball and disc may result in the agglomeration of debris, promoting abrasive wear and increasing the wear rate. Nevertheless, as the sliding velocity intensifies, the debris becomes partially scattered from the contact area and partially relocated on the counter material surface. This process leads to the creation of a transfer layer that lowers the wear rate.

The rate of wear demonstrates a proportional increase with the duration of sliding across all samples. Additionally, a rise in the wear rate is evident as the glass fiber content decreases. These findings align with the conclusions drawn by Kukureka et al. [24] in their study on rolling–sliding contact involving a similar material.

The wear rate of the sample with 54 wf % GF exhibited a more pronounced sensitivity to sliding speed compared4 to composites with higher weight percentages when subjected to a normal load of 10 N. This wear pattern arises from alterations in the characteristics of the interacting surfaces as the GF content decreases within the PA matrix. Figure 11 and Table 7 illustrate that a lower normal load and reduced speed have a minimal impact on the wear rate of all tested samples.

A significant gap is observed when the normal load is increased by 30 N, and sliding speed is around 0.25 m/s especially for the reinforcement rate of 54 wf %. 

It was found that larger asperities on the metal counterface deform the polymer surface, giving rise to plowing, micro cutting, and the formation of abrasive wear tracks.

The wear observed in reinforced samples containing glass fibers is a result of the abrasive impact of metal asperities. Additionally, the abrasive action is intensified as particles of glass debris combine. Usually, the diameter of GF undergoes reduction before fracturing into shorter lengths, influenced by the alternating stress system, and subsequently experiences debonding from the polymer matrix. 

GF can have both abrasive and lubricating effects on the sliding interface, and their role in reducing friction, especially during the run-in period, is multifaceted. A more detailed explanation of how glass fibers can contribute to both abrasive and friction-reducing effects is, the glass fibers are hard and rigid and in the initial stages of sliding, they can come out of the polymer matrix and mechanically abrade the steel ball as seen in Figure 14. This leads to abrasive wear of the steel surface, resulting in the generation of wear debris and the removal of material from the counterface.

As the glass fibers abrade in contact with the ball, wear particles are generated. These wear particles are composed of both glass fibers and metal particles. The presence of these particles leads to abrasive wear mechanisms during the break-in period. Paradoxically, the wear residues generated by the abrasion of the glass fibers act as a form of lubrication. Smaller wear particles act as solid lubricants or fillers, reducing direct contact between sliding surfaces and decreasing friction. This effect is particularly prominent during the break-in period, when the surfaces adapt to each other.

Glass fibers contribute to the formation of transfer films on the surface material. Transfer films are thin layers of material transferred from one surface to another during sliding. This oxide acts as solid lubricants, providing a protective layer that reduces friction. Glass fibers, as they break down, can be involved in creating these transfer films.

On the other hand, the mechanical breakdown of the glass fibers during the lapping period can lead to the production of smaller particles. These particles may initially act as abrasive wear but may also contribute to the smoothing of surface asperities. Smoother surfaces experience reduced friction due to reduced adhesion and contact area.

During the running period, the tribological system undergoes adaptive changes as the surfaces interact and conform to each other. Glass fibers, as they wear and undergo mechanical changes, contribute to these adaptations. These changes can lead to improved conformability between the polymer matrix and the counterface, leading to a reduction in friction.

## 5. Design of Experiments

The results obtained for the 27 samples that were tested considering the control parameters presented in Table 7 are encompassed in the same table for each sample. The purpose of the experimental work was to offer a basis for determining the sample with the optimal material recipe. The parameters that were considered to affect the dry sliding wear and friction process were the applied load, the sliding speed, and the fiber content. The ELECTRE multicriterial decision making method was used to determine the optimal parameters considering multiple performance characteristics. 

**Figure 15:**
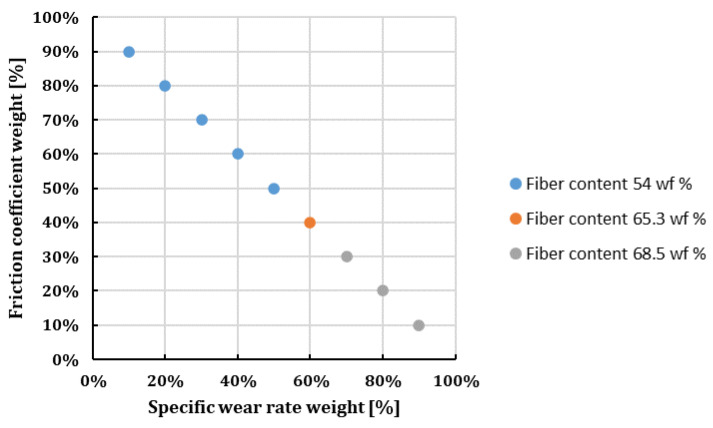
Optimal options with respect to different criteria weight combinations (F = 10 N; v = 0.1 ms^−1^).

First, the matrix of values (see Section 2.4) was generated according to Table 7. Then, the utilities for each sample were computed using Equations (1) and (2) considering the specific wear rate as a minimum criterion (“lower-the-better”) and the friction coefficient as a maximum criterion (“higher-the-better”). The two considered criteria were assigned weights that varied between 10% and 90% (with their sum being 100%), representing the importance of each criterion for the person who must decide which material recipe to use. For each criteria weight combination with the sum 100%, the concordance and discordance indices were determined based on Equations (3) and (4) from Section 2.4. The difference between the two types of indices allowed the classification of the samples with respect to the chosen criteria and their weights. After performing the classification for each weight combination, the third sample (with an applied load of 10 N, a sliding speed of 0.1 ms^−1^ and a fiber content of 54 wf %) was determined to be amongst the first four best options for material recipes for all weight combinations, thus making it the optimal one. A detailed presentation of the optimal material recipe samples with respect to the weight combination is given in Figure 15, where all three presented samples were tested at an applied load of 10 N and a sliding speed of 0.1 ms^−1^.

## 6. Conclusions

It is important to note that the specific reasons can vary depending on the type of composite, the nature of the sliding contact, and the environmental conditions. The observed decrease in the coefficient of friction might be a result of a combination of these factors, therefore detailed experimental analysis and characterization are typically required to understand the specific mechanisms at play in each composite material. 

In this investigation, the mechanical and tribological properties of three GFRP composites were examined. The purpose of the experimental work was to offer a basis for determining the sample with the optimal material recipe.

The combination of polymers with GF can offer a balance of properties suitable for various applications, especially when low forces and low peripheral speeds are involved.

The findings indicated that augmenting the weight fraction of GF resulted in enhanced strength properties, including elastic modulus, flexural modulus, ultimate strength, and flexural strength, while decreasing ductility (elongation at break). With an increase in the GF in wf %, the coefficient of friction reduced in the ball-on-disc contact configuration. Abrasive friction mechanisms were evident for GFRP. As the GF weight fraction rose, the wear rate increased in the ball-on-disc contact configuration. This increase in the wear rate until K = 32.73 × 10^−5^ mm^3^ × (Nm)^−1^ was particularly notable at higher loads, specifically 20 N, sliding speed of 0.25 ms^−1^ and more pronounced for higher GF contents in weight percentage (65.3 wf %).

The optimal parameter combination identified using the ELECTRE method in the current experimental research is denoted as F_1_v_1_C_3_ signifying an applied load of 10 N (F_1_), a sliding speed of 0.1 ms^−1^ (v_1_) and a composite with 54 wf % fiber content (F_3_). The worn surface exhibits fractures and damage on the matrix, plateau formation, fiber-matrix pull-out, fiber pull-out, and a variety of wear mechanisms. These were mostly caused by design factors including applied force, sliding speed, and wf % of the fibers.

It is important to note that the specific application requirements, environmental conditions, and the mechanical properties needed should be considered when selecting materials for a particular use case. The combination of polymers with GF can offer a balance of properties suitable for various applications, especially when low forces and low peripheral speeds are involved.

## Figures and Tables

**Table 1:** Flexural properties of GFRP discs.

Specimen Type	Flexure Stress (MPa)	Flexure Strain (%)	E-Modulus (MPa)
GFRP 68.5%	415.5 (21.5)	3.1 (0.13)	18,218 (392)
GFRP 65.3%	388.6 (37)	2.4 (0.25)	21,009 (484)
GFRP 54%	464.8 (11.5)	3.8 (0.16)	14,935 (199)

**Table 2:** Tensile properties of GFRP discs.

Specimen Type	Tensile Strength (MPa)	Tensile Strain at Tensile Strength (mm/mm)	E-Modulus (MPa)
GFRP 68.5%	480.1 (26)	2.9 (0.15)	22,182 (253)
GFRP 65.3%	376.57 (24.5)	2.6 (0)	18,721 (316)
GFRP 54%	301.8 (18)	2.85 (0)	12,602 (301)

**Table 3:** Hardness and roughness (R_a_) value for GFRP samples.

Specimen Type	C_1_-GFRP 68.5%	C_2_-GFRP 65.3%	C_3_-GFRP 54%
Hardness HR-30Tscale (MPa)	47.1	41.5	22.4
Equivalent HRB scale (MPa)	46	38	11
Roughness R_a_ (µm)	0.37	0.34	0.069

**Table 4:** Mechanical properties of the steel ball sample [4].

Ball Type (12.7 mm)	Hardness HRC Scale	Compressive Strength (MPa)	Yield Strength (MPa)	Young’s Modulus (GPa)	Poisson’s Ratio	Roughness R_a_ (µm)
52100 Chrome Alloy Steel ρ = 7.81 g/cm^3^	54–58	2100–2200	2000	200	0.3	0.282–0.30

**Table 5:** Matrix of values.

Criteria	Cr_1_	Cr_2_	...	Cr_j_	...	Cr_n_
Weights	w_1_	w_2_	...	w_j_	...	w_n_
Options						
O_1_	o_11_	o_12_	...	o_1j_	...	o_1n_
O_2_	o_21_	o_22_	...	o_23_	...	o_2n_
...			...		...	
O_i_	o_i1_	o_i2_	...	o_ij_	...	o_in_
...			...		...	
O_m_	o_m1_	o_m2_	...	o_m3_	...	o_mn_

**Table 6:** Control factors and their levels of dry sliding wear.

Factor	Description	Level I	Level II	Level III
F	Normal load (N)	10	20	30
v	Sliding velocity (m s^−1^)	0.1	0.25	0.36
C	Fiber content (wt%) *	68.5%	65.3%	54%

* Note: starting now, in the paper, the GFRP disc with 68.5% fiber content will be marked with C_1_, the one with 63.5% with C_2_, and the last one with 54% with C_3._

**Table 7:** Experimental results of dry sliding wear.

Experimental Parameters			Optimizing Parameters		
Exp.no.	Applied Load F (N)	Sliding Speed v (ms^−1^)	Fiber Content C (wt%)	Specific Wear Rate K [10^−^⁵ mm^3^ × (Nm)^−1^]	Coefficient of Friction (µ) Average of the Last 60 min
1	10	0.1	68.5	1.5488	0.43
2	10	0.1	65.3	3.42	0.5
3	10	0.1	54	5.758	0.54
4	10	0.25	68.5	6.4267	0.38
5	10	0.25	65.3	12.2667	0.39
6	10	0.25	54	20.985	0.4
7	10	0.36	68.5	9.59	0.34
8	10	0.36	65.3	16.0216	0.38
9	10	0.36	54	14.90746	0.33
10	20	0.1	68.5	11.456	0.41
11	20	0.1	65.3	14.1887	0.48
12	20	0.1	54	15.208	0.54
13	20	0.25	68.5	6.12	0.42
14	20	0.25	65.3	32.737	0.43
15	20	0.25	54	24.906	0.28
16	20	0.36	68.5	4.42	0.35
17	20	0.36	65.3	23.291	0.36
18	20	0.36	54	26.0193	0.37
19	30	0.1	68.5	14.4255	0.48
20	30	0.1	65.3	18.2557	0.49
21	30	0.1	54	21.6711	0.51
22	30	0.25	68.5	14.34102	0.33
23	30	0.25	65.3	23.727	0.36
24	30	0.25	54	32.3849	0.37
25	30	0.36	68.5	10.4919	0.47
26	30	0.36	65.3	19.1906	0.48
27	30	0.36	54	14.488	0.49

## Data Availability

All the data are available with the authors and can be provided on request. Correspondence and requests for materials should be addressed to C.B., M.C. and F.S. The data are not publicly available until M.C. publishes his PhD thesis.
